# Effects of self-avatar cast shadow and foot vibration on telepresence, virtual walking experience, and cybersickness from omnidirectional movie

**DOI:** 10.1177/20416695241227857

**Published:** 2024-02-22

**Authors:** Junya Nakamura, Yasushi Ikei, Michiteru Kitazaki

**Affiliations:** 13129 Toyohashi University of Technology, Japan; 13143 The University of Tokyo, Japan; 13129 Toyohashi University of Technology, Japan

**Keywords:** walking, omnidirectional movie, cast shadow, avatar, tactile perception

## Abstract

Human locomotion is most naturally achieved through walking, which is good for both mental and physical health. To provide a virtual walking experience to seated users, a system utilizing foot vibrations and simulated optical flow was developed. The current study sought to augment this system and examine the effect of an avatar's cast shadow and foot vibrations on the virtual walking experience and cybersickness. The omnidirectional movie and the avatar's walking animation were synchronized, with the cast shadow reflecting the avatar's movement on the ground. Twenty participants were exposed to the virtual walking in six conditions (with/without foot vibrations and no/short/long shadow) and were asked to rate their sense of telepresence, walking experience, and occurrences of cybersickness. Our findings indicate that the synchronized foot vibrations enhanced telepresence as well as self-motion, walking, and leg-action sensations, while also reducing instances of nausea and disorientation sickness. The avatar's cast shadow was found to improve telepresence and leg-action sensation, but had no impact on self-motion and walking sensation. These results suggest that observation of the self-body cast shadow does not directly improve walking sensation, but is effective in enhancing telepresence and leg-action sensation, while foot vibrations are effective in improving telepresence and walking experience and reducing instances of cybersickness.

Locomotion is a critical aspect of virtual environments (VEs) and numerous techniques for achieving it have been proposed (Di Luca et al., 2021). Unlike in the real world, locomotion in VEs is not subject to the same physical laws and constraints on body motion. As such, a range of approaches suited to various purposes have been developed, including teleportation (Bozgeyikli et al., 2016; Christou & Aristidou, 2017), game controller-based locomotion (Cardoso, 2016; Nabiyouni et al., 2015), virtual vehicles (Bruder et al., 2012; Medeiros et al., 2019; Ware & Osborne, 1990), gestural controls (McCullough et al., 2015; Ouzounis et al., 2015; Terziman et al., 2010), and walking (Iwata, 1999; Medina et al., 2008; Razzaque et al., 2002).

The walking experience involves multimodal sensations as well as active movements of the legs and arms. Self-motion perception from optic flow and vestibular sensation, and haptic perception of the feet are representative sensations during walking. In this study, we defined the virtual walking experience as the subjective sensations of walking, including self-motion perception and leg actions in the VE.

Virtual walking presents the challenge of simulating the feeling of walking without or with minimal physical movement, and a variety of equipment and techniques have been developed to achieve this goal (Freiwald et al., 2020; Ikeda et al., 2004; Ikei et al., 2015; Kallioniemi et al., 2017; Kaneko et al., 2018; Kitazaki et al., 2019; Kokkinara et al., 2016; Lécuyer et al., 2006; Matsuda et al., 2021; Nakamura et al., 2021; Saint-Aubert et al., 2023; Terziman et al., 2012; Turchet et al., 2012). These techniques often involve the presentation of sensory stimuli such as vision (Ikeda et al., 2004; Lécuyer et al., 2006), audition (Rodger et al., 2013; Turchet, 2016), tactile sensation (Matsuda et al., 2021; Nakamura et al., 2021; Terziman et al., 2012), and proprioception through motion (Freiwald et al., 2020; Kaneko et al., 2018) that are similar to those encountered during actual walking. Multimodal systems that incorporate various stimuli (Ikei et al., 2015; Turchet et al., 2012) are also used. Turchet et al. (2012) developed a virtual walking system that combines visual scenes, footstep sounds, and vibrations on the feet, while Ikei et al. (2015) developed the five-sense theater (Five Star) system which passively induces proprioception through actuators, along with tactile sensation, vision, audition, and smell.

The perception of self-motion through visual motion in a large visual field is known as vection (Dichgans & Brandt, 1978; Palmisano et al., 2015; Riecke, 2011). This phenomenon is frequently employed in VEs to generate a sense of locomotion. Vection can be induced through motion presented in a large visual field (Dichgans & Brandt, 1978; Riecke & Jordan, 2015), with an emphasis on background rather than foreground movement (Brandt et al., 1975; Ohmi et al., 1987), and non-attended rather than attended motion (Kitazaki & Sato, 2003). It has also been shown to be enhanced through the use of perspective jitter (Palmisano et al., 2000; 2003) and realistic textures (Riecke et al., 2005).

In virtual reality (VR), Lécuyer et al. (2006) utilized simulated camera motion, or perspective jitter, to improve the sensation of walking. In addition to vection, it is also possible to induce a virtual walking sensation through the conveyance of tactile sensation to the feet (Kitazaki et al., 2019; Terziman et al., 2012). For example, Terziman et al. (2012) enhanced the walking sensation by combining camera motion, foot vibration, and footstep sounds.

Omnidirectional movies are often utilized in virtual walking systems. Ikeda et al. (2004) developed a system that projects images onto a multiscreen display and combines it with a treadmill, while Kallioniemi et al. (2017) compared the immersion provided by a multiscreen display to that of a head-mounted display (HMD). Nakamura et al. (2021) proposed a system that utilizes synchronized foot vibrations in conjunction with omnidirectional movies, which was found to also enhance the perception of ground material when suitable vibration patterns were used for various scenes.

In this study, presence or telepresence is defined as the psychological experience of feeling present in a VE, a critical factor in achieving a positive VR experience (Heater, 1992; Minsky, 1980). VR allows users to feel as if they are in the VE, even though they are actually in a different and real place (Sadowski & Stanney, 2002; Weech et al., 2019). The sense of presence involves not only the perception of being “there,” but also influenced by users’ virtual body (avatar) and body movements (Grabarczyk & Pokropski, 2016; Slater & Steed, 2000; Slater & Usoh, 1993a; Slater et al., 1995b). Avatars are more than mere digital representations; they are mediums through which users project themselves into the VE (Slater et al. 2009; Slater & Usoh 1993b; Waltemate et al. 2018). These avatars allow users to embody a presence in VEs, intensifying the sense of being there. Waltemate et al. (2018) showed that personalized avatars greatly affect body ownership, presence, and emotional response in VEs, indicating how avatar customization directly impacts user experience and presence. Creating avatars that closely mimic real movements and appearances contributes to a high level of embodiment and, as a result, a deeper sense of presence (Gonçalves et al., 2022).

Matsuda et al. (2021) found that the virtual walking experience could be enhanced when one observes their avatar from a first-person perspective through a virtual mirror. In their study, participants seated in the real world observed their virtual avatar walking in the VE. The virtual mirror within the environment enabled participants to view their avatar as though they were looking at their own reflection. This stimulus fostered a more robust connection between the participant's physical body and their virtual avatar. As a result, observing the self-avatar improved subjective scores for walking sensation, leg action sensation, and telepresence in the VE. Kokkinara et al. (2016) showed that a walking virtual avatar in a first-person perspective not only enhanced the sensation of walking, but also induced feelings of body ownership, agency, and self-localization in the avatar's location while participants were seated in a real location, compared to observation in a third-person perspective. As indicated in the previous paragraph, embodiment, which refers to a sense of body ownership, agency, and self-localization, facilitates telepresence in a physical or VE (Grabarczyk & Pokropski, 2016; Haans & IJsselsteijn, 2012). Thus, experiencing walking, embodiment, and telepresence are closely related.

Given these contexts, we chose telepresence as one of our dependent variables because telepresence is linked to the walking sensation and embodiment in addition to the immersion in VEs. In this study, we use the term telepresence because it refers to the feeling of being present in a remote or VE, while the term presence has a broad meaning that includes the feeling of being present in the real environment.

HMDs typically have a narrower viewing angle than the actual field of vision, leading to a lack of visual information on the self-body in a first-person view. To address this, Matsuda et al. (2021) used mirrors in a VE. While artificial mirrors can be useful in experimental settings, they may also be perceived as unnatural objects that do not exist in real life, potentially causing discomfort for users. The incorporation of mirrors into 3D models and omnidirectional movies using real-world textures is also challenging. As such, we focused on the use of cast shadows as a means of enhancing the perception of the self-body without introducing discomfort.

Cast shadows are formed when one surface occludes another from a light source (Mamassian et al., 1998), and the human visual system is able to quickly distinguish cast shadows from attached shadows and detect discrepancies in illumination (Elder et al., 2004). Valid cast shadows have been shown to facilitate visual search, indicating that they are rapidly identified and discounted by an early-level visual system (Rensink & Cavanagh, 2004). They are effective for the discrimination of object shape (Mamassian et al., 1998; Norman et al., 2000) and for the perception of object shape and size (Yonas et al., 1978), although their efficacy for object recognition is somewhat controversial (Braje et al., 2000; Castiello, 2001). In particular, cast shadows are advantageous for the perception of the relationship between an object and its background, including three-dimensional relative positions (Madison et al., 2001; Mamassian et al., 1998). The three-dimensional motion trajectory of an object can also be modulated by the motion of its cast shadow (Kersten et al., 1996, 1997), an effect that has been observed in infants as young as 6 months old (Imura et al., 2006). The presentation of cast shadows has also been shown to modulate motor control in a reaching task (Bonfiglioli et al., 2004).

The cast shadows of the body can elicit an automatic orientation of attention towards the self-body, promote the binding of personal and extrapersonal space (Pavani et al., 2014; Pavani & Castiello, 2004; Pavani & Galfano, 2015), and embody one's own body (Kuylen et al., 2014). Galfano and Pavani (2005) also found that body shadows function as a hint to direct tactile attention. Cast shadows have been shown to increase presence in VR (Slater et al., 2009, 1995a), with Slater et al. (1995a) reporting that they enhance presence for visually dominant individuals in a first-person VE. The incorporation of cast shadows and reflections into visually realistic scenes may also lead to more realistic behavioral responses (Slater et al., 2009). The blending of object shadows into augmented reality (AR) environments has been found to enhance the presence of objects (Sugano et al., 2003). The length of shadow can influence human perception and navigation in VEs, and the long shadow is better than the short shadow (Sugano et al., 2003; Slater et al., 1995a). Kwon et al. (2015) found that artificially generated shadows of virtual objects projected onto the floor improved depth perception in a 3D stereoscopic environment. Adams et al. (2022) pinpointed that the presence of a cast shadow influenced depth perception in AR displays. In summary, cast shadows have been shown to contribute to the perception of object position, depth, shape, and action, as well as presence. On the other hand, humans were insensitive to the lighting inconsistency that could be detected by the direction of cast shadows (Ostrovsky et al. 2005). However, their impact on the virtual walking experience remains uncertain.

Cybersickness, or VE sickness, is a significant problem in VR. The sensory conflict theory, which posits that motion sickness is caused by a conflict between vision and proprioception (or vestibular sensation), is the most widely accepted explanation for this phenomenon (Oman, 1990; Reason, 1978). When users see moving scenery but do not receive accurate or consistent vestibular sensations, this can lead to sensory conflict and subsequently to cybersickness. To decrease this visual-vestibular conflict, there are two main approaches: minimizing or blurring the field of view to reduce visual information during motion (Budhiraja et al., 2017; Fernandes & Feiner, 2016), or improving vestibular information through the presentation of tactile sensations (Liu et al., 2019; Plouzeau et al., 2017; Weech et al., 2018). In walking on an omni-directional treadmill, cybersickness varies with changes in the speed of movement (Lohman & Turchet, 2022). Based on this, we predicted that synchronized foot vibrations, in conjunction with optic flow during virtual walking, could reduce instances of cybersickness.

Sensory feedback mechanisms, such as vibrotactile stimuli and visual cues, play an important role in modulating users’ experiences and perceptions in VEs. The work of Lee et al. (2017) highlighted how vibrotactile feedback, such as vibrations, can enhance the sense of social presence with a virtual counterpart. Complementarily, Rosa et al. (2015) showed that visual cues, particularly those related to weight, have the potential to alter the perception of vibrotactile sensations, suggesting an underlying interaction between the visual and tactile sensory modalities. Feng et al. (2016) extended this finding by investigating the synergy of multisensory feedback on user experience, where they examined the sound and vibration of footsteps, wind, and vision on user performance and experience. Collectively, these studies point to the intricate interaction between different sensory inputs in shaping perception in virtual spaces. Building on these findings, it is imperative to further explore the possible interactions between specific independent variables in our study, namely shadow length and foot vibrations. The interplay between visual and tactile cues in VEs remains a topic of considerable interest in psychological research. In particular, it is crucial to determine whether the perceptual influence of foot vibrations is modulated by the existence and length of a shadow cast within the VE.

## Research Purposes and Hypotheses

The present study aimed to contribute to the further development of the virtual walking system for a seated observer without leg movements (Kitazaki et al., 2019; Matsuda et al., 2021; Nakamura et al., 2021). The synchronous foot vibration with optic flow during walking enhances the virtual walking experience and telepresence compared to the asynchronous or no vibration (Kitazaki et al., 2019). One's own avatar observed in first-person perspective through mirrors improves virtual walking sensations (Matsuda et al., 2021). However, the effect of the avatar's cast shadow on the walking experience, telepresence, and cybersickness has not been investigated, and the impact of foot vibrations on cybersickness remains unexplored. We aimed to clarify these issues.

### Effects of Cast Shadows on Telepresence and Virtual Walking Experience

The first purpose of this study was to determine whether it is possible to enhance the virtual walking experience through the construction of an environment that synthesizes an omnidirectional movie and a self-body avatar, presenting the shadow of the avatar as a hint of the self-body's existence. We compared the effects of long, short, and no shadows of the avatar on the telepresence and the virtual walking experience. We assumed that the long shadow could be more effective than the short shadow because the long shadow would be more noticeable than the short shadow. We hypothesized that observing the body's cast shadow would increase telepresence in the VE (H1-a), and observing the shadow is posited to improve the virtual walking experience (H1-b).

### Role of Foot Vibrations in Telepresence and Virtual Walking Experience

We sought to replicate the effect of foot vibrations on telepresence and walking experience. We hypothesized that foot vibrations would improve telepresence in the VE, consistent with previous studies (Kitazaki et al., 2019; Matsuda et al., 2021; Nakamura et al., 2021) (H2-a). In addition, we hypothesized that foot vibrations would also enhance the virtual walking experience (H2-b).

### Impact of Cast Shadows and Foot Vibrations on Cybersickness

While this study primarily focused on enhancing the virtual walking experience through shadows and vibrations, we also considered the impact these factors might have on user comfort, specifically in terms of cybersickness. Specifically, we hypothesized that the incorporation of an avatar's cast shadows could influence the level of cybersickness experienced by participants in the VE (H3-a). In addition, foot vibrations, which serve as a sensory feedback mechanism, may also play a distinct role in reducing the intensity of cybersickness in the virtual walking context (H3-b). These hypotheses stem from the understanding that consistent and realistic sensory feedback in VEs can lead to reduced sensory conflict, which is often a primary cause of cybersickness.

## Methods

### Stimuli and Conditions

#### Visual Stimuli

**Figure 1.**
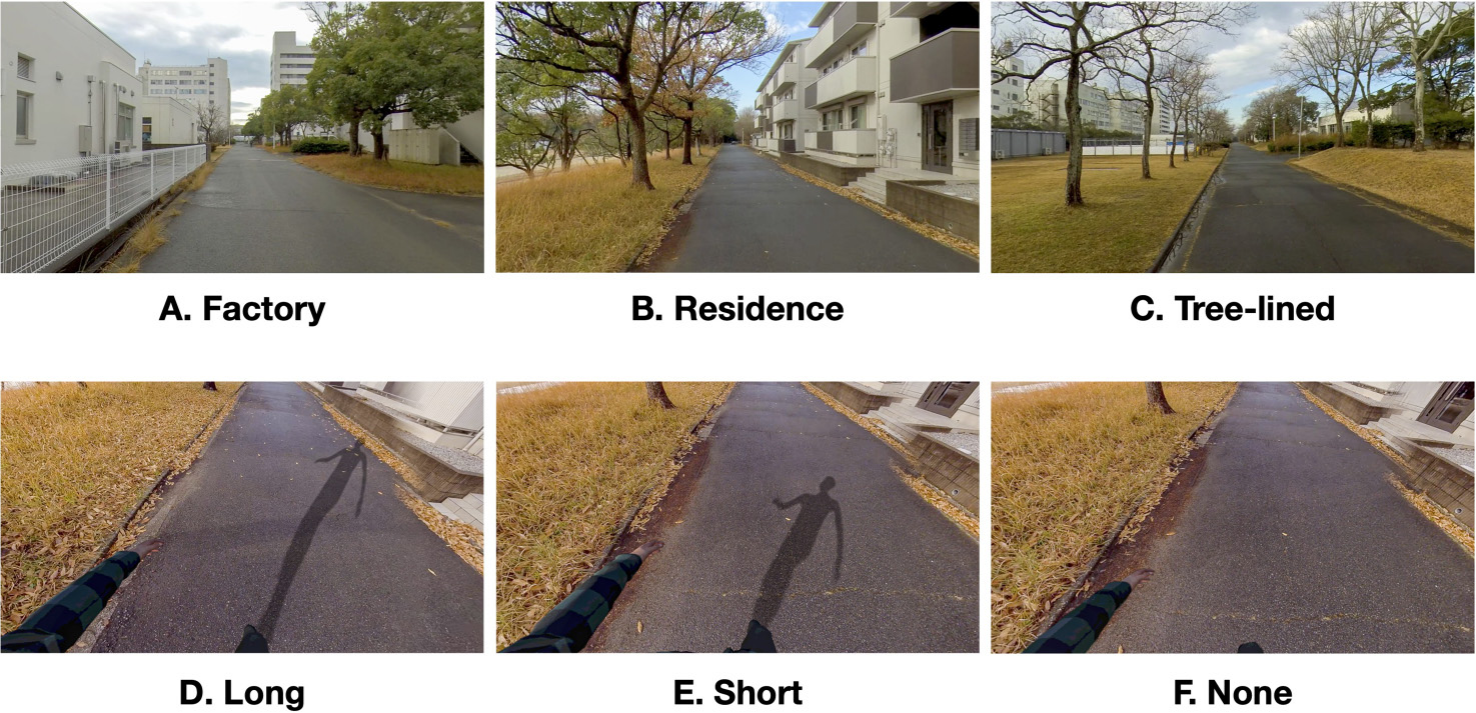
Example images of omnidirectional movies (A, B, C) and shadow conditions (D, E, F) in visual stimuli. (A) Passing scene by the factory. (B) Passing scene by the residence. (C) Passing scene of a tree-lined street. (D) Long shadow. (E) Short shadow. (F) No shadow.

Three omnidirectional movies with different locations were used for visual stimuli. The road surfaces in the three videos were all asphalt pavements (Figure 1A to C). The reason for preparing three kinds of videos is to prevent mental saturation by repetition of a single kind of videos.

All the videos were taken in the campus of Toyohashi University of Technology. All videos were captured with an omnidirectional camera (Insta360 Pro 2, 7680 [width] × 3840 [height] pixels, 60 fps). All scenes were forward-moving at a nearly constant speed (1.55–1.62 m/s) for 60 s in the experiment. The image stabilization was applied to the video to reduce the oscillation of the video. The weather at the capturing/shooting was cloudy and shadowless. This type of weather was chosen so that the real shadows and the artificial shadows used in the experiment would not conflict.

Omnidirectional movies were drawn inside an ICO sphere placed on Unity. A body avatar was attached, and walking animation (FinalIK; Walk, 120–124 steps / min) synchronized with each video was applied.

The cast shadow of the avatar was drawn by the light source (Directional Light) in the position of left rear 30 degrees. For the height of the light source, the condition (Toyohashi University of Technology, 2022/1/1, suncalc.org) of the moving image photographing place was set. As shadow conditions, three types of long shadow (3:00 p.m.), short shadow (12:00 p.m.), and no shadow were set (Figure 1D to F).

We used Unity's Cubemap to capture the shadow and avatar in the video. After capturing, the cubemap was converted to 360-degree equirectangular format in real-time and then rendered on another transparent ICO sphere. The transparent ICO sphere with the shadow and avatar was set inside of the movie rendered ICO sphere. The center of the ICO spheres was made to be the viewing point of the HMD, so that the participants observed the scene with shadow and avatar from the first-person viewpoint of the avatar. Only the rotation of the HMD was reflected in the scene. The visual display was presented in monocular vision (identical images for both eyes). Participants can observe the entire area by changing their posture, allowing them to directly observe the view, the avatar, and the avatar's shadow.

#### Tactile Stimuli

**Figure 2.**
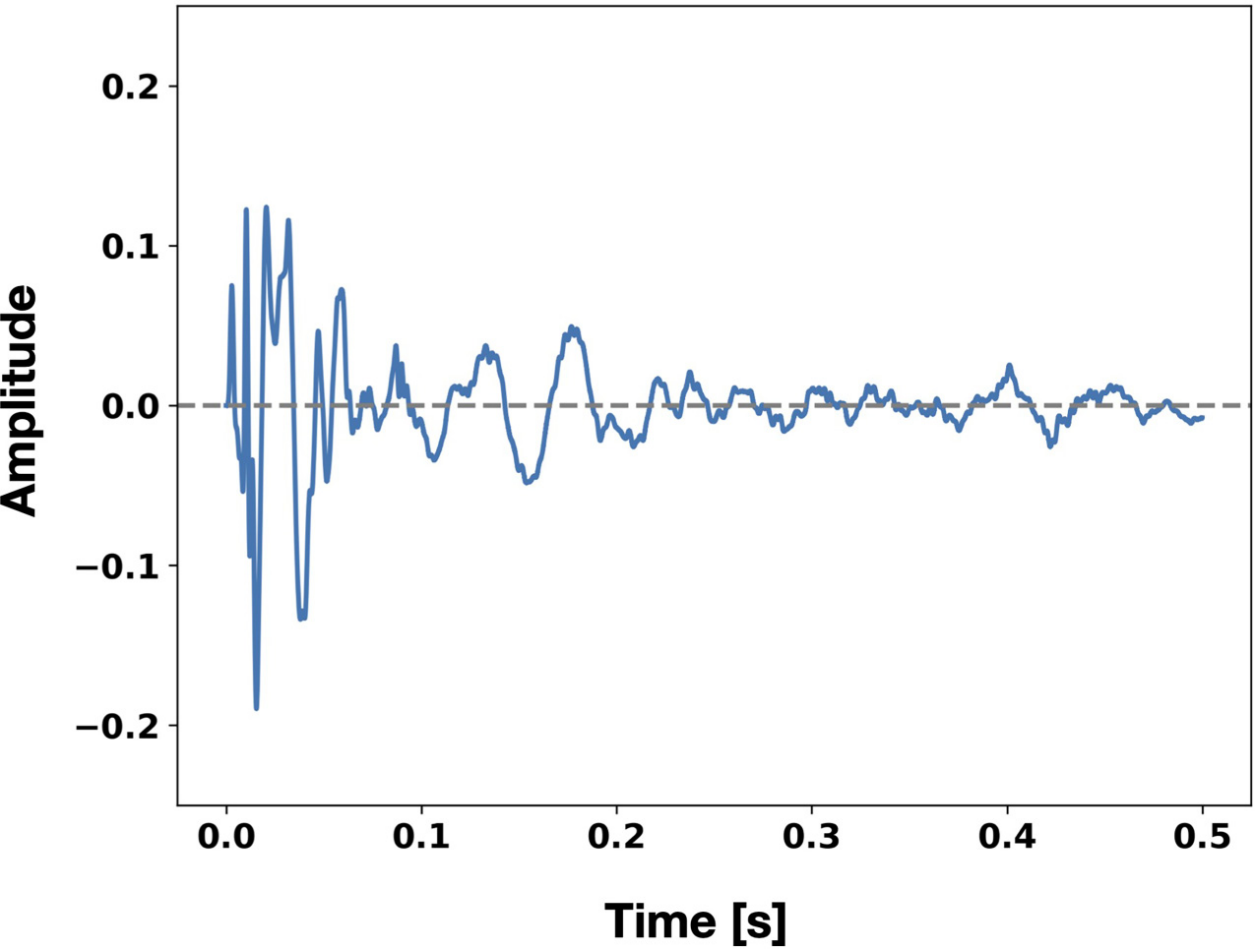
Amplitude waveforms of vibro-tactile stimuli.

Vibro-tactile stimuli were presented to the heel and forefoot of the left and right feet. These stimuli were made by recording the sound of walking in sneakers at the video recording site. A 500 ms sound clip including the vibration decay was created by cutting a single step from the recorded data and applying a 400 Hz low-pass filter to present only the vibration component (Figure 2). The same sound clip was used for the heel and forefoot.

These stimuli were synchronized with the landing timing of the heel of the walking avatar, and vibration of the forefoot was presented after the heel. The difference in vibration timing between the heel and forefoot was set to 100 [ms] because the load response period in the walking cycle occupied about 10 [%] of the whole walking cycle (Perry & Burnfield, 2010).

The walking speed depended on the omnidirectional movie and was synchronized with the avatar's foot movements (1.55–1.62 m/s).

#### Conditions

The experimental conditions were combinations of three different shadow conditions (long, short, no-shadow) and two vibration conditions (on, off) with within-subject design. The participants observed 18 trials (three shadow conditions × two vibration conditions × three locations of videos) in random order. The location factor was for repetition and merged in the following analysis.

### Participants

Twenty volunteers participated in the experiment. The participants were all male, mean 21.15 years old with 1.65 standard deviation (SD). The mean height of the participants was 172.5 cm ± 4.21 SD. The sample size was determined by a power analysis: a medium effect size *f* = 0.25, alpha = 0.05, power = 0.8, and repeated measures of analysis of variance (ANOVA), three shadow conditions × two vibration conditions, using G*Power 3.1 (Faul et al., 2007; 2009). This sample size is comparable to that of previous studies that have effectively detected medium to large effects (Matsuda et al., 2021; Nakamura et al., 2021). All participants had normal binocular vision and physical abilities. They gave written informed consent before the experiment. The experiment was approved by the Ethical Committee for Human-Subject research at Toyohashi University of Technology and was performed in accordance with the committee's guidelines and regulations.

### Apparatus

**Figure 3.**
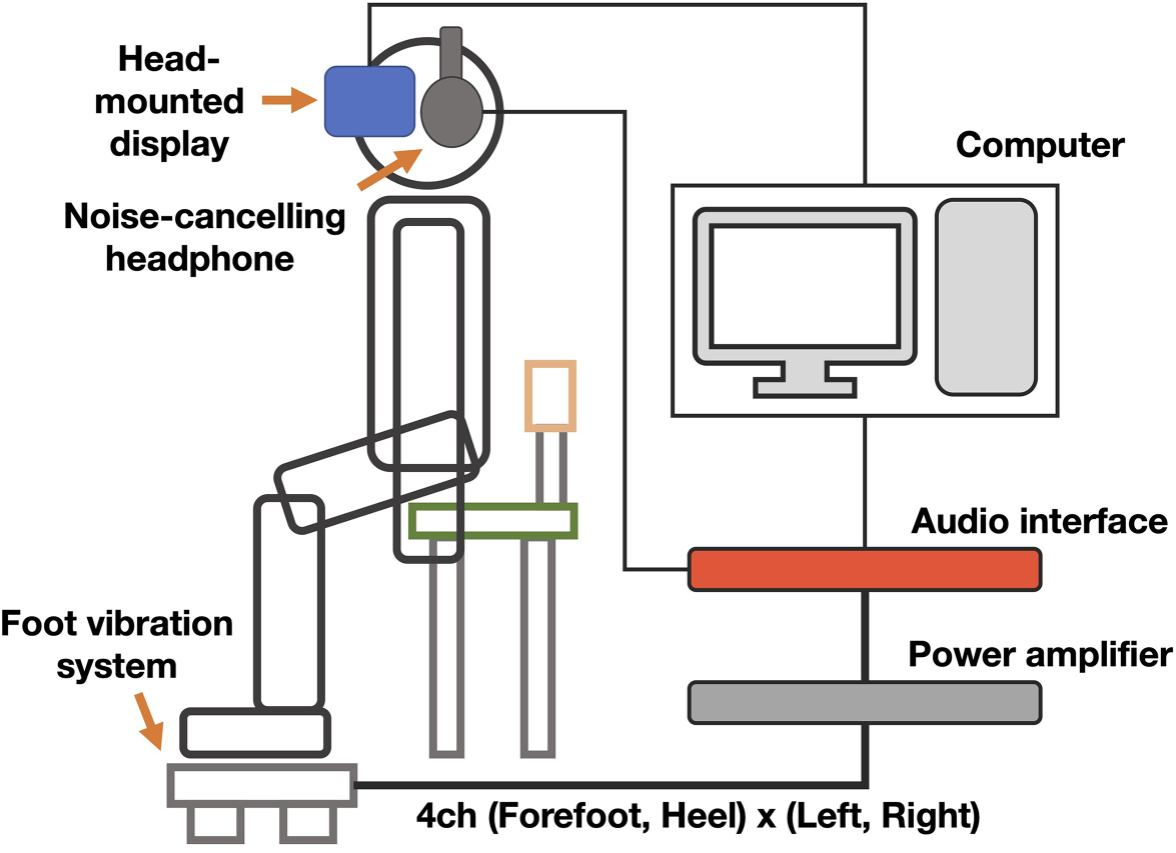
Overview of the virtual walking system. The participants sat on a chair during the experiment. Visual stimuli were presented on an HMD controlled by a computer, which also controlled and presented tactile stimuli through an audio interface and amplifier, driving the vibration system. Noise-canceling headphone was used in conjunction with white noise to mask the sound from the vibration system.

A computer (Intel Core i7 10700, NVIDIA GeForce RTX 2070 Super, DDR4 32 GB) was used for the experiment, and the presented stimuli were controlled by Unity (2020.3.20f1). The participants sat on a chair, wore a HMD and a pair of headphones, and placed their feet on a foot vibrator (Figure 3).

The visual stimulation was presented by the HMD (HTC Vive Pro Eye, 1440 [width] × 1600 [height] pixels for each eye, refresh rate of 90 Hz).

Tactile stimuli were presented to the forefoot and heel of the left and right feet with four vibro-transducers (Acouve Lab Vp408, 16–15,000 Hz).

**Figure 4.**
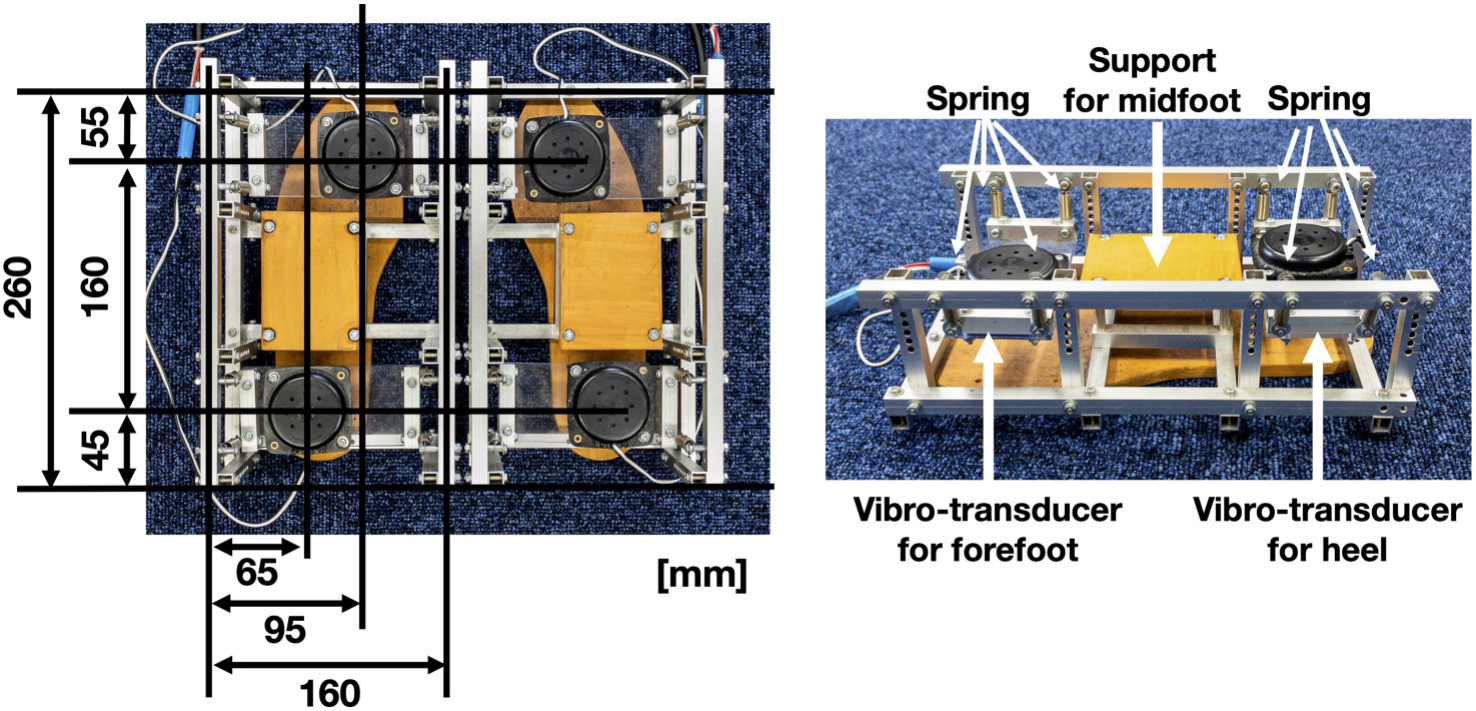
The foot vibration system. Four vibration transducers presented vibrations on the left and right forefeet and heels. The vibro-transducers were spring-suspended from the aluminum frame to prevent vibration from transmitting to other components.

These transducers were mounted on an acrylic plate separately and suspended with four springs on each forefoot and heel in an exterior composed of an aluminum frame, while the midfeet of participants were supported by wood plates that were connected to the frames (Figure 4).

The transducers were driven by a multichannel power amplifier (Behringer EPQ304, 40 W × 4 / 8Ω) and an audio interface (Behringer FCA1616), and ASIO is used for the driver interface. The amplitude of vibration was fixed throughout the experiment and was strong enough with all participants wearing socks.

The participants wore headphones (SONY WH-1000XM4) to prevent participants from hearing auditory stimuli generated by the transducer and the external environment. Headphone noise cancellation was used during stimulus presentation, and white noise (70 dBA) was presented to mask the external sounds.

### Questionnaire

**Figure 5.**
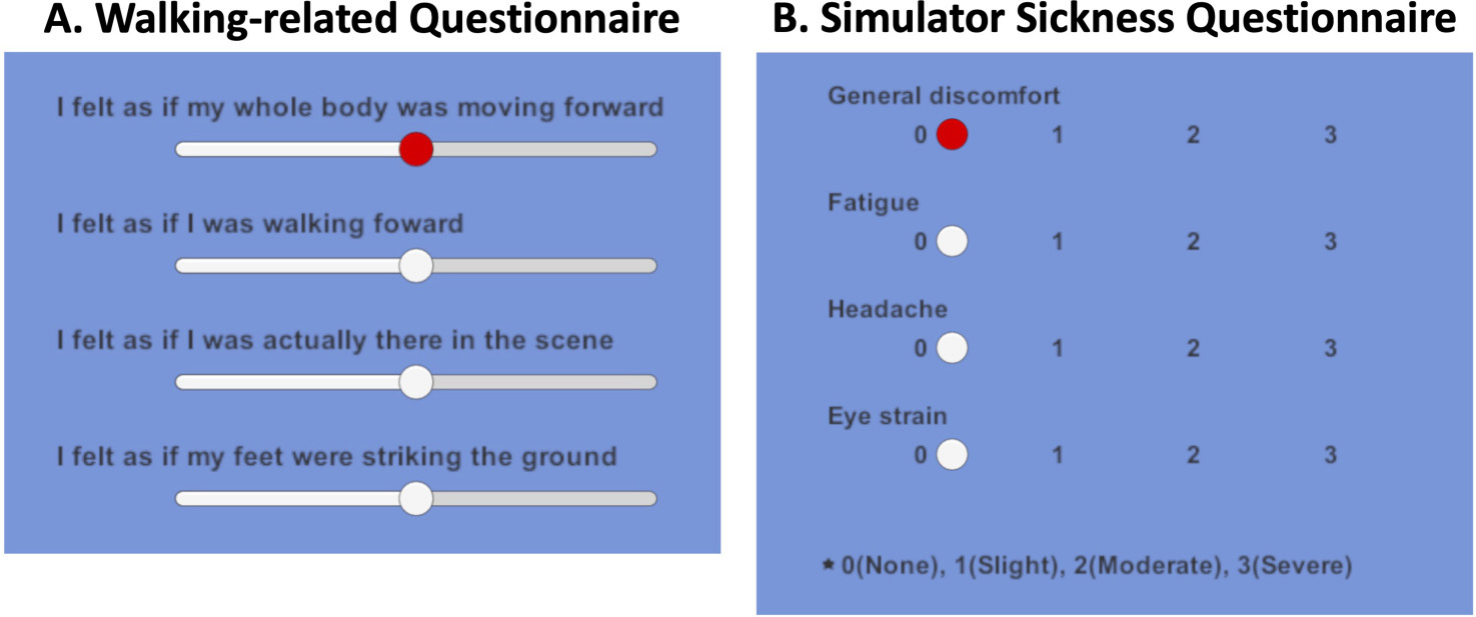
Two questionnaires. Participants were asked to respond to each item on a visual analog scale (VAS). The order of items on each questionnaire was randomized in each trial. (A) Example of response screens for questionnaire items on telepresence and walking experience. (B) Example of the answer screen for the Simulator Sickness Questionnaire.

Subjective ratings for telepresence and walking experience were obtained after stimulus observation (Figure 5A). The rating items were based on previous studies (Kitazaki et al., 2019; Matsuda et al., 2021; Nakamura et al., 2021). The walking experience had three items. The order of these items was randomized in each trial.

I felt as if I were actually there in the scene (telepresence).

I felt as if my whole body was moving forward (self-motion).

I felt as if I was walking forward (walking sensation).

I felt as if my feet were striking the ground (leg action).

The responses of participants were obtained using a visual analog scale. A line was presented on a screen, and the leftmost side of the line implied no sensation, whereas the right side of the line implied the same sensation as in the actual walking experience. The data were digitized from 0 to 100 for the analysis. They then completed the Simulator Sickness Questionnaire (SSQ) (Kennedy et al., 1993) with 16 items at four levels (Figure 5B).

### Procedure

The subjects sat on a chair, placed their feet on sole vibrators, and wore an HMD and headphones. Each trial consisted of a blank screen (5 s), a fixation cross (5 s), and a stimulus presentation (60 s). At the beginning of the stimulus presentation part, the participants looked around the surroundings and were made to recognize the scenery, the self-body avatar, and its cast shadow. After observation, the evaluation on the walking and the SSQ were performed. The participants performed a total of 18 trials (3 levels of shadow conditions × 2 levels of vibration conditions × 3 locations) in random order.

### Statistical Analysis

For the telepresence and walking experience data, the ratings in three locations of videos were averaged. The evaluation data of SSQ was converted into Total Score, and three sub-scores (Nausea, Oculomotor, Disorientation). Statistical tests were performed with the R 4.1 and JASP 0.16.3 software.

First, both data were tested for normality using the Shapiro-Wilk test (α = 0.05). If the data did not violate the normality (*p* > .05), a two-way repeated measures ANOVA was performed. If the data violated the normality test (*p* < .05), a two-way repeated measures ANOVA was performed with an aligned rank transformation (ANOVA with ART) procedure (Wobbrock et al., 2011) as a non-parametric test. Then, we performed an analysis of simple main effects and a post-hoc multiple-comparison analysis as necessary.

The Bayesian factors were calculated for all parametric tests. The Bayes factor indicates the strength of evidence for H_1_ compared to H_0_. According to Kass & Raftery (1995), BF_10_ of 1 to 3.2 are considered weak evidence, BF_10_ of 3.2 to 10 are substantial evidence, and BF_10_ above 10 are strong evidence.

For parametric data, if there was a lack of sphericity with Mendoza's multisample sphericity test, the reported values were adjusted using the Greenhouse–Geisser correction (Geisser & Greenhouse, 1958). Shaffer's modified sequentially rejective Bonferroni procedure was applied for post-hoc multiple comparisons. For non-parametric data, Tukey's method with Kenward–Roger degrees of freedom approximation (Kenward & Roger, 1997) was applied for post-hoc multiple comparison analysis.

## Results

For the telepresence and walking-related data, the leg-action responses were significantly deviated from the normality, whereas the others did not violate the normality. For the SSQ data, all scores were significantly deviated from the normality. Thus, ANOVA with ART was conducted for non-parametric data and ANOVA for others (self-motion, walking, and telepresence). We show the score for telepresence followed by three sets of scores for walking experience.

### Telepresence

**Figure 6.**
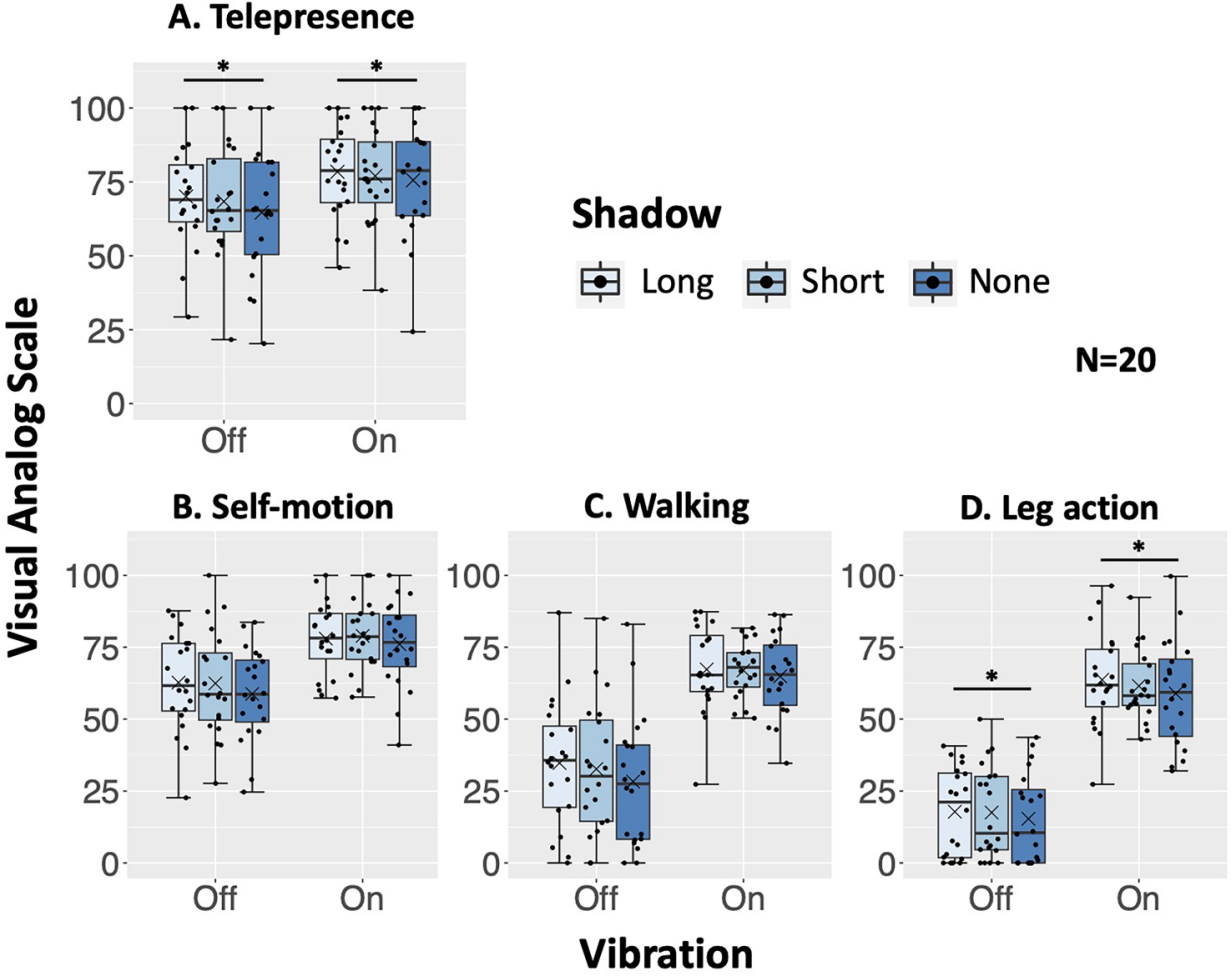
Results of telepresence (A) and walking experience (B, C, D): (A) telepresence, (B) self-motion sensation, (C) walking sensation, (D) leg action sensation. The horizontal axis indicates the condition of vibration. The vertical axis indicates the digitized visual analog scale (VAS) values (0 to 100). A box indicates the range between Q1 (25%) and Q3 (75%), the thick line in the box indicates the median, and a set of whiskers indicates the maximum and minimum values. The dots indicate the dispersion of scores, and the cross (×) indicates the mean value. *p < .05.

For the telepresence score (Figure 6A), the ANOVA revealed a significant main effects for the vibrations [F(1, 19) = 23.156, *p* = .0001, η_p_^2 ^= 0.549, BF_10_ = 238.998], and shadow condition [F(2, 38) = 4.630, *p* = .016, η_p_^2 ^= 0.196, BF_10_ = 1.229]. The interaction was not significant. The scores were higher in the vibration condition than in the no-vibration condition, irrespective of the shadow conditions. The post-hoc analysis of the shadow conditions showed that the scores for the long-shadow condition were significantly higher than the no-shadow condition [t(19) = 2.899, adj.*p* = .028, BF_10_ = 4.253].

The long shadow and foot vibration increased the telepresence over the no-shadow and the no-vibration conditions, supporting the hypotheses H1-a and H2-a.

### Walking Experience

#### Self-Motion Sensation

For the self-motion sensation (Figure 6B), the ANOVA revealed a significant main effects for the vibrations [F(1, 19) = 26.037, *p* < .0001, η_p_^2^= 0.578, BF_10_ = 237.08]. The scores were significantly higher in the vibration condition than in the no-vibration condition, irrespective of the shadow conditions. Either the main effect of shadow condition [F(2, 38) = 2.767, *p* = .076, η_p_^2 ^= 0.127, BF_10_ = 0.634] or the interaction was not significant.

#### Walking Sensation

For the walking sensation (Figure 6C), the ANOVA revealed a significant main effects for the vibrations [F(1, 19) = 55.507, *p* < .0001, η_p_^2^= 0.745, BF_10_ = 2.256 × 10^4^]. The scores were higher in the vibration condition than in the no-vibration condition, irrespective of the shadow conditions. Either the main effect of shadow condition [F(2, 38) = 1.794, *p* = .180, η_p_^2^= 0.086, BF_10_ = 0.334] or the interaction was not significant.

#### Leg-Action Sensation

For the leg-action sensation (Figure 6D), the ANOVA with ART revealed a significant main effects for the vibrations [F(1, 19) = 111.46, *p* < .0001, η_p_^2^= 0.854], and shadow condition [F(2, 38) = 4.005, *p* = .026, η_p_^2^= 0.174]. The interaction was not significant. The scores were higher in the vibration condition than in the no-vibration condition, irrespective of the shadow conditions. The post-hoc analysis of the shadow conditions showed that the scores for the long-shadow condition were significantly higher than the no-shadow condition [t(19) = 2.760, adj.*p* = .023].

#### Summary of Walking Experience

The rhythmic foot vibration was dominant in all scores for walking experience regardless of the shadow condition. The long shadow increased the leg-action sensation over the no-shadow condition, but did not significantly affect the self-motion and walking sensations. These results supported the hypotheses H2-b, and did not support H1-b.

### Simulator Sickness Questionnaire

**Figure 7.**
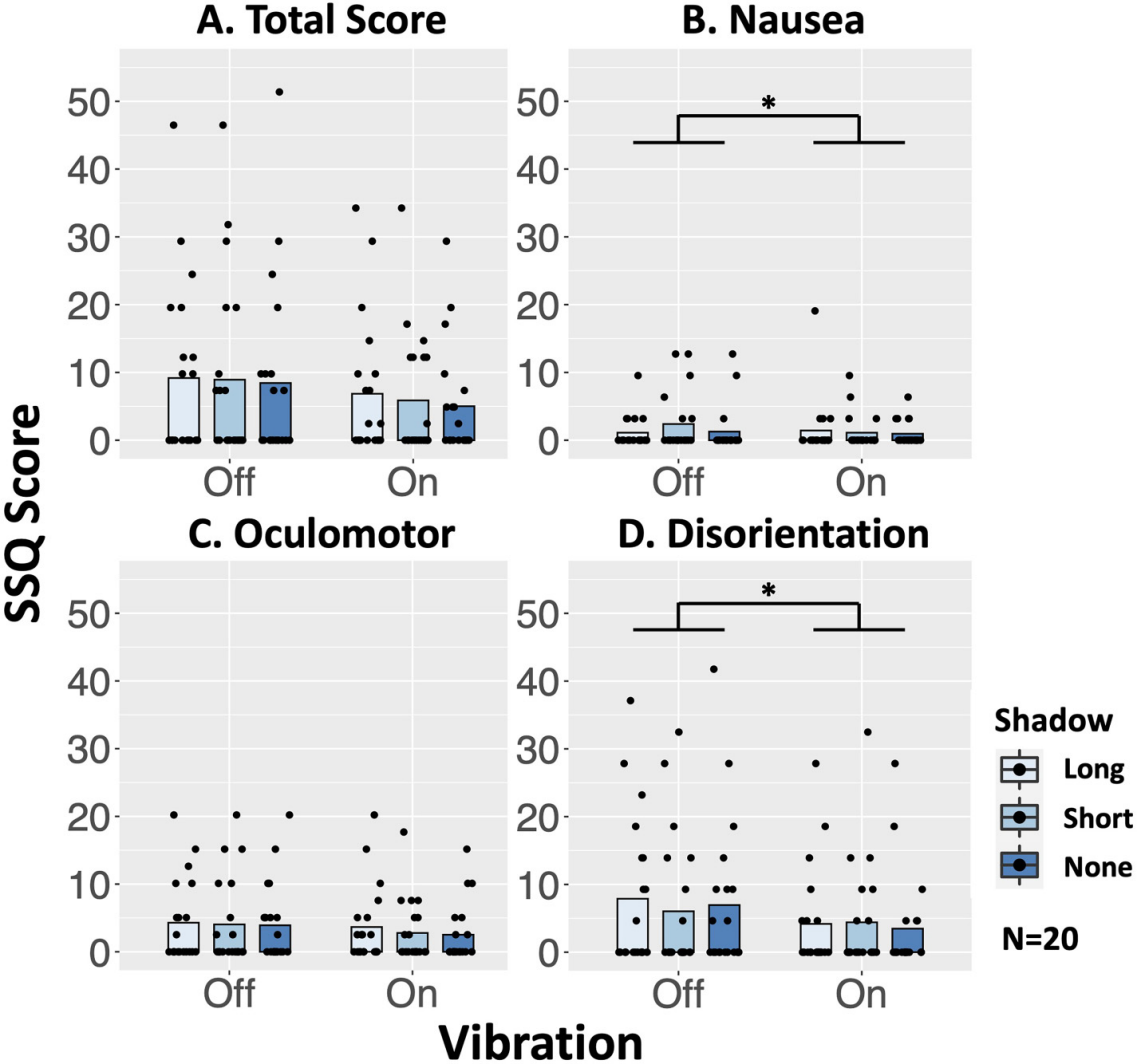
Results of simulator sickness questionnaire score: (A) total score, (B) nausea, (C) oculomotor, (D) disorientation. The horizontal axis indicates the condition of vibration. The vertical axis indicates the SSQ score. The bar indicates the average score of the SSQ, and the dots indicate the scores distribution. *p < .05.

SSQ data did not satisfy the normality in all items. Total and Oculomotor scores did not show a main effect of either Vibration or Shadow, or those interaction (Figure 7A and C).

For the Nausea score (Figure 7B), the ANOVA with ART revealed a significant main effects of the vibrations [F(1, 19) = 19.220, *p* = .0003, η_p_^2^= 0.503], and the interaction [F(2, 38) = 8.366, *p* = .0009, η_p_^2^= 0.306]. The main effect of the shadow condition was not significant. The main effect of the vibration indicated that the Nausea score was significantly decreased by the foot vibration. However, a simple main effect analysis did not show significance in any shadow conditions [Long-Shadow: F(1, 19) = 0.237, *p* = .632, η_p_^2^= 0.0123; Short-Shadow: F(1, 19) = 1.829, *p* = .192, η_p_^2^= 0.088; No-Shadow: F(1, 19) = 0.560, *p* = .463, η_p_^2^= 0.029] and vibration conditions [Off: F(2, 38) = 2.347, *p* = .109, η_p_^2^= 0.110; On: F(2, 38) = 0.156, *p* = .856, η_p_^2^= 0.008]. The post-hoc multiple comparisons analysis of the simple effects revealed no significant effect for all avatar conditions and vibration conditions combinations.

For the Disorientation score (Figure 7D), the ANOVA with ART revealed a significant main effects for the vibrations [F(1, 19) = 8.834, *p* = .0078, η_p_^2^= 0.317]. Either the main effect of the shadow condition or the interaction was not significant. The Disorientation score was significantly decreased by the foot vibration [t(19) = 2.972, adj.*p* = .078].

These results partially supported the hypothesis H3-b, but did not support H3-a.

## Discussion

### Summary of Results

We investigated whether the cast shadow of a self-avatar could enhance the virtual walking experience in a first-person perspective. We found that observing the cast shadow enhanced sensations of leg-action and telepresence (H1-a), but did not significantly impact sensations of self-motion or walking (H1-b). This supports H1-a but does not support H1-b. Synchronized foot vibrations, on the other hand, enhanced sensations of self-motion and walking (H2-a), as well as leg-action and telepresence (H2-b), regardless of the presence of a cast shadow. This supports both H2-a and H2-b. The cast shadow had no effect on the cybersickness (H3-a). Foot vibrations decreased nausea and disorientation (H3-b), but had no significant effect on other scores on the SSQ, including the total score and oculomotor score. These results partially support H3-b, but did not support H3-a.

### Effect of Cast Shadow on Telepresence (H1-a)

The effects of the cast shadow of a self-avatar were limited. While the cast shadow increased the sensations of leg action and telepresence, it did not affect the sensations of self-motion or walking. Therefore, H1-a (that the observation of the body's cast shadow would increase presence in the VE) was supported by the telepresence results. Since the cast shadow enhances the perception of 3D scenes and telepresence (Adams et al., 2022; Kwon et al., 2015; Slater et al., 1995a; Sugano et al., 2003), the telepresence in our virtual walking system was enhanced by the long cast shadow as well. The short shadow cast may be less noticeable to participants and have no effect on the telepresence. We speculate that the effect of cast shadows on telepresence may be positively correlated with the contrast and realism of the shadows, unless the shadows are too unnatural.

### Effect of Cast Shadow on Walking Experience (H1-b)

The long cast shadow improved the sensation of leg action, but did not improve the sensation of self-motion or walking. Thus, H1-b (that the virtual walking experience would be enhanced by the cast shadow) was not supported by the results. The cast shadow of an object has been shown to contribute to the perception of the object's shape and size, as well as to clarify the spatial relationship between the object and its background (Mamassian et al., 1998; Yonas et al., 1978). Therefore, we speculate that the cast shadow of a self-avatar might contribute to the perception of the shape and motion of the observer's legs, which are not visible due to the limited field of view of the HMD, and thus enhance only the leg motion perception.

When the distance between the cast shadow and the object changes, the perception of the object's position and motion is also modulated (Kersten et al., 1996). In the present study, however, the cast shadow was always attached to the self-avatar, so that self-motion perception was not modulated by the cast shadow. These results suggest that leg action perception may be partially independent of walking perception, while walking perception is more closely related to self-motion perception.

It can be argued that the cast shadow served to increase the intensities of the vibration stimuli and to increase the ratings of the leg action sensations. We should measure perceived foot vibration intensities to test this argument in a future study.

### Effects of Foot Vibrations on Telepresence Walking Experience (H2-a, H2-b)

Rhythmical foot vibrations simulating the avatar's walking clearly enhanced sensations of self-motion, walking, leg-action, and telepresence in all shadow conditions. This confirms both H2-a and H2-b (that foot vibrations enhance walking experiences), replicating the findings of previous studies (Kitazaki et al., 2019; Matsuda et al., 2021; Nakamura et al., 2021; Terziman et al., 2012). The integration of foot vibrations was effective in improving both the perception of walking and the sense of presence in the VE, consistent with our hypotheses.

### Effects of Cast Shadows on Cybersickness (H3-a)

The presence of an avatar's cast shadows did not notably influence levels of nausea or disorientation, failing to support H3-a (that cast shadows impact cybersickness in VEs). This absence of a significant effect contrasts with findings from Ng et al. (2018), who argued that matching visual-motion stimuli effectively mitigated cybersickness, aligning with the Sensory Conflict Theory (Oman, 1990; Reason, 1978). However, our study failed to find such an effect, possibly because the scenes chosen were less likely to induce motion sickness, focusing on walking experiences. This explanation is in line with Pouke et al. (2018), who found only weak evidence that increased visual realism in the form of detailed graphics influences cybersickness. This suggests that visual cues like shadows may not be potent enough to significantly reduce sensory conflict and subsequent cybersickness.

Moreover, individual differences may also be at play in our observations. Ling et al. (2013) highlighted that factors such as immersive tendencies and monocular visual ability are correlated with the extent to which individuals experience presence and cybersickness in VEs. Hence, the variability in our participants’ responses could also be influenced by such individual characteristics. Given the contrast between our findings and established theories like the Sensory Conflict Theory, further studies are warranted to explore the effects of visual cues and individual characteristics on cybersickness in a variety of virtual settings.

### Effects of Foot Vibrations on Cybersickness (H3-b)

The rhythmical foot vibrations decreased nausea and disorientation, partially supporting H3-b (that foot vibrations decrease VE sickness). This may be due to the reduced discrepancy between optical flow and walking experience caused by the foot vibrations. Previous studies have also found that foot vibrations can reduce cyber sickness (Plouzeau et al., 2017), and our results support these findings. However, the effect of seat vibrations on visually induced motion sickness remains controversial (D'Amour et al., 2017; Jung et al., 2021; Plouzeau et al., 2017; Sawada et al., 2020). In our study, we presented vibrations on the feet that were synchronized with walking in the scene, while a previous study (Jung et al., 2021) used seat vibrations to simulate a motorcycle engine. It is possible that walking is more familiar to participants and the relationship between the vibrations and the walking is clearer in our study, making foot vibrations more effective at reducing motion sickness. However, the effect was not significant on the total or oculomotor scores of the SSQ.

This may be because the experimental scenes were chosen to be less likely to cause motion sickness in order to investigate walking experiences, leading to relatively low levels of sickness. Cybersickness during observing of 2D omnidirectional movie could be different from standard 3D VR environments (Kasahara et al., 2015; Shahid Anwar et al., 2020). Therefore, a further research is needed to investigate the effectiveness of foot vibrations simulating walking on VE sickness in various situations.

### Theoretical and Practical Contributions

The present study contributes to the research field by introducing a novel approach to enhance the virtual walking experience for seated users. The combination of foot vibrations and synchronized optical flow with an avatar's cast shadow was designed to enhance the sensations of self-motion, walking, leg action, and telepresence during virtual walking. We found that the foot vibrations enhanced all sensations, but the cast shadow enhanced only the sensations of leg action and telepresence. Thus, the haptic stimulation of the feet is critical for both the walking experience and telepresence, while the cast shadow of the avatar is critical only for telepresence.

We investigated the effect of the augmented system on cybersickness. The results provide valuable insights into how the use of synchronized foot vibrations can reduce instances of nausea and disorientation sickness during virtual locomotion. It is theoretically consistent with the sensory conflict theory of sickness.

This research has practical implications for the design and improvement of VR systems, particularly those involving virtual locomotion. The findings of this study can be used to design more immersive and less uncomfortable VEs for seated observers by presenting foot vibrations and the shadow cast by the avatar. Thus, the virtual walking system could provide realistic walking experiences and sensations presented in VEs without cybersickness to people with mobility impairments.

### Limitations

The foot vibrations were generated from footstep sounds by cutting high frequency components, resulting in tactile stimulation that was not identical to real tactile sensations during walking. In future studies, it would be important to develop more appropriate tactile stimulation. Three types of movies were used, but all of them were on concrete paved roads, and only one type of vibration was used. Therefore, they were essentially the same environment. All optic flow and tactile vibrations from the video were pre-recorded and subjects had no control over them. Therefore, the effect of participants’ active gait control and interactive navigation with the environment should be investigated in the future.

The omnidirectional scene in the current study was two-dimensional and lacked binocular disparity. The self-avatar and its cast shadow were composed in a three-dimensional environment using Unity, but the entire scene was presented without binocular disparity in order to maintain consistency. The use of a three-dimensional omnidirectional scene could potentially enhance the walking experience. The artificial cast shadows used in this study may have caused a discrepancy with the lighting in the omnidirectional movies, potentially leading to a conflict in scenery perception. Using more realistic and consistent cast shadows may enhance their effects on the virtual walking experience.

To gain a more comprehensive understanding of the virtual walking experience, future research should also consider using more sophisticated questionnaires and objective measures for assessing walking experience and telepresence. In the current experiment, participants may guess the aims of study or the hypotheses and respond in the questionnaire, although the study procedure was designed to minimize disclosure of the experimental hypothesis. The lack of procedures or objective measures to minimize such demand characteristics is a limitation of this study.

Moreover, examining the ecological validity of the setup, including factors such as the speed of motion and shadow shape, is important to ensure that the illusion of self-motion and walking are maintained. Finally, including more test participants and assessing their imaginary capabilities, such as their kinesthetic motor imagery scores and external or internal visual motor imagery (Kooijman et al. 2022; Väljamäe et al. 2008), could provide insight into which manipulations have a stronger perceptual salience.

Finally, the generalizability of the study's findings is limited by the use of single-item measures for each variable. Although this strategy streamlined the experimental design, it sacrificed the depth of information that could have been obtained by implementing multi-item scales. This limitation should be considered in a future study.

### Conclusion

This study examined the effects of a virtual walking environment synthesized from an omnidirectional movie and a self-body avatar with its cast shadow. Foot vibrations synchronized with the video and avatar's walking were presented at the forefeet and heels. The avatar's cast shadow enhanced the sense of telepresence and leg-motion, while the foot vibrations improved telepresence as well as self-motion, walking, and leg-action sensations, and reduced nausea and disorientation sickness scores. The avatar and shadow synthesis technique used in this study can be applied to a range of omnidirectional movies, potentially allowing for the conversion of existing movies into virtual walking experiences that may contribute to improving the mental health of individuals with mobility limitations.
